# Sinonasal adenoid cystic carcinoma following formaldehyde exposure in the operating theatre

**DOI:** 10.1186/s12995-014-0043-4

**Published:** 2014-12-17

**Authors:** Anniken Sandvik, Tor Audun Klingen, Sverre Langård

**Affiliations:** Department of Environmental and Occupational Medicine, Oslo University Hospital, PO Box 4956, Nydalen, Oslo, NO-0424 Norway; Department of Pathology, Vestfold Hospital Trust, Tønsberg, Norway

**Keywords:** Sinonasal cancer, Adenoid cystic carcinoma, Formaldehyde, Work-related cancer, Nurse, Operating theatre

## Abstract

We present a case report of an auxiliary nurse who developed an adenoid cystic carcinoma in her left maxillary sinus following occupational exposure to formaldehyde in the operating theatre. Currently, the epidemiological evidence that formaldehyde can cause cancer in humans is considered to be limited. Previous case-control-studies of formaldehyde and sinonasal cancer have mainly investigated subjects who were concomitantly exposed to wood dust, a known risk factor to the development of sinonasal adenocarcinoma of intestinal type. Our case report presents a patient who has developed an adenoid cystic carcinoma following exposure to formaldehyde. We suggest that the occupational physician remains alert to formaldehyde as an occupational hazard among health care workers.

## Background

Cancers of the nasal cavity and paranasal sinuses occur with low incidence, with annual incidence rates less than 1 per 100 000 (i.e. 1x10^−5^/y) in most countries [1]. The etiology is often related to exposure at work and an increased risk is well established among furniture workers and other woodworkers [2]. The latency period from exposure to wood dust until presentation of a respiratory tract malignancy has been found to be approximately 40 years [2]. An elevated risk has also been observed among workers in nickel refineries, textile industries, chromate pigment manufacture, and boot- and shoe industries [3]. Recently, it has also been suggested that work-related exposure to formaldehyde may enhance the risk of sinonasal cancers, in particular adenocarcinomas [4]. Experimental animal studies support the notion that inhalation of formaldehyde is carcinogenic [5]. Long-term exposure to high levels of formaldehyde may occur during varnishing of wood, finishing of textiles, and in certain other industrial settings, whereas high short-term exposure has been reported for embalmers, pathologists and paper workers [6]. A pooled analysis investigated the association between occupational exposure to formaldehyde and sinonasal cancer from 12 case-control-studies [4]. This study showed an increased risk for adenocarcinoma in both men and women, also in the subjects who were thought to never have been exposed to wood or leather dust. Against these positive findings, however, no excess of mortality from sinonasal cancer has been observed in cohort studies of formaldehyde-exposed workers [6,7].

In hospitals, formaldehyde is widely used to preserve tissue samples, and chemical disinfectants containing formaldehyde have been extensively used in the cleaning of operating theatres [8]. We report on a case of adenoid cystic carcinoma in the left maxillary sinus in an auxiliary nurse exposed to formaldehyde during her work in the operating theatre.

Adenoid cystic carcinoma (ACC) is a rare salivary gland-type adenocarcinoma of unknown etiology. The most common sites of ACC appear to be the minor salivary glands of the oral cavity and the major salivary glands, but 10–25% occurs in the sinonasal tract [9]. In this location, they are thought to originate from seromucus glands of the nasal cavity and paranasal sinuses as well as the surface epithelium. Following squamous cell carcinomas, ACC and adenocarcinomas of non-salivary gland-type (intestinal or non intestinal) are the malignant sinonasal tumors with highest incidence [10,11]. ACC accounts for approximately 10% of all malignancies in the sinonasal tract [12]. The tumor is composed of myoepithelial cells with cribriform (sieve-like), tubular and solid growth pattern in a myxohyaline stroma. Perineural invasion is very common, and invasion of major (ie, cranial) nerves is a predictor of poor prognosis. Symptoms may include nasal obstruction, epistaxis, and pain, simulating inflammatory conditions, and this can lead to a delay in diagnosis and treatment. These tumors can attain large sizes with extensive infiltrative growth in the surrounding bone (skull base) at presentation, and margin status is a significant predictor of outcome [10]. The lung is the most common site of distant metastasis, followed by bone, liver and brain [13]. A recent meta-analysis of sinonasal ACC showed a 5-year overall survival of 62% [14].

## Case presentation

**Figure 1:**
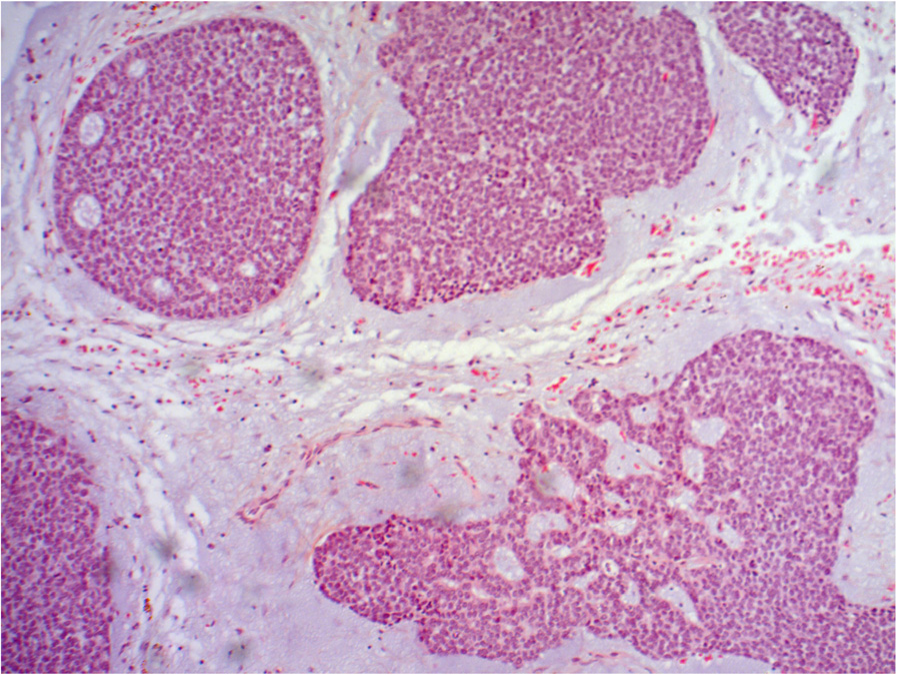
**Adenoid cystic carcinoma of the patient.** The image shows islands of fairly uniformly sized tumor cells with solid growth pattern and partly small cribriform spaces. The epithelial component is sharply demarcated from the myxoid interstitial stroma. The image is photographed at 400 times magnification.

A 72-year old woman experienced numbness below her left eye. She was examined with a CT scan of the paranasal sinuses revealing a tumor in the left maxillary sinus. Subsequently, exploratory surgery was carried out with tissue sampling of the mucosa in the floor of the sinus. The tumor was histologically classified as an adenoid cystic carcinoma. Immunohistochemical staining was positive for coloring with CD117 (C-kit). This supports the classification as an adenoid cystic carcinoma. Special coloring with Alcian also showed positive and strong coloring in the stroma between the cell layers. This further supports the diagnosis adenoid cystic carcinoma. The tumor did not show perineural invasion. A microscopical image of the tumor is presented in Figure 1. The patient was treated with radiation therapy intended to cure.

Her medical history included hypercholesterolemia, macular degeneration, psoriasis and a long history of Sjögren’s syndrome. Because of dry mouth and severe caries due to Sjögren’s syndrome, the patient had had all her teeth removed and she had tooth implants in her lower jaw and dentures in her upper jaw. She had smoked 10 cigarettes a day for 45 years.

Prior to the onset of her present disease, she had worked as an auxiliary nurse at a local hospital for 31 years. For 11 years, in the period 1967–1988, she had been working exclusively in the operating theatre at the general surgical ward. Her work tasks during this period had involved the handling of biopsies and she put the tissue samples in containers filled with formaldehyde. Containers of various sizes were employed: most surgeries involved preserving smaller tissue samples, but some surgeries involved preservation of larger organs, e.g. whole breasts. She had handled tissue samples several times per day throughout the 11 year period. Larger tissue samples were handled approximately a couple of times a week. After use, our patient disposed of the formaldehyde by pouring it into the sink in the operating theatre. Her work tasks also included preparing the surgical patients by cleaning the skin with a solution of iodine in ethanol and assisting as a non-sterile-assistant at various surgeries. In addition, her work involved daily disinfection of the operating theatre after use. The disinfection was performed by spraying the walls of the theatre, a procedure lasting 1 or 2 hours, which was performed three or four times a day throughout her 11 year period of work in the surgical ward. For the rest of her career, she had worked as an auxiliary nurse in the medical ward of the hospital, without any known exposure to formaldehyde.

Disinfectants for use in hospitals may contain formaldehyde [8]. Although the patient did not remember what kind of disinfectant that was used at her work-place, it is possible that the patient was exposed to formaldehyde from disinfectant in addition to the formaldehyde used for the preservation of tissue samples. Throughout her 11 years of work in the operating theatre, she did not use adequate airway protection to prevent inhalation of formaldehyde. To our knowledge, measurements of formaldehyde in her work athmosphere were never performed. Both surgeons and nurses may have been exposed to formaldehyde in the operating theatres of the hospital. However, by directly handling the formaldehyde for tissue samples and for disinfection of the operating theatre, our patient was conceivably exposed to higher levels of formaldehyde vapors than her fellow workers. Her cancer was diagnosed 45 years after the first known exposure to formaldehyde vapors.

## Conclusions

We report on a patient who had developed an adenoid cystic carcinoma in a paranasal sinus following exposure to formaldehyde at her work place during the years 1967–1988. The time period from the first exposure until the cancer was diagnosed was 45 years. This is consistent with the latency period of approximately 40 years seen in the development of respiratory tract cancers following exposure to wood dust [2]. The International Agency for Research on Cancer (IARC) has concluded that there is only limited epidemiological evidence that formaldehyde can cause sinonasal cancer in humans [6,7]. The observation that almost all formaldehyde-exposed case subjects in the available case-control studies [4] had also been exposed to wood dust was commented upon, and it was argued that this may have resulted in an erroneously high relative risk, in particular for adenocarcinoma [6,7]. The present case report may illustrate a possible association between formaldehyde exposure and sinonasal cancer without concomitant exposure to wood dust. It should however, be noted that our patient presented with an adenoid cystic carcinoma. This rare type of salivary gland adenocarcinoma is not known to be associated with the exposure to wood dust such as is the case with intestinal type adenocarcinoma and to some extent squamous cell carcinoma. The etiology of adenoid cystic carcinoma is considered to be mainly unknown. However, environmental and work exposure to factors such as rubber manufacturing, hair dressers, beauty shops and nickel compounds have been reported to be associated with the development of salivary gland tumors [15]. We do not know of any other reports in which the development of adenoid cystic carcinoma is associated with exposure to formaldehyde. Therefore we find it important to report on this patient.

We do not have access to measurements of the levels of formaldehyde exposure at our patient’s work place. The estimation of exposure in her case is based solely on her work history and her recollection of the working conditions. From studies performed in histopathology laboratories, it has been found that concentration of formaldehyde can be high during tissue disposal and preparation of formalin [16]. The usual mean concentration during exposure in this setting is approximately 0,5 ppm [6]. From a German study, the mean exposure level during disinfection of operating theatres was measured to be 0,8 ppm [8]. Although the exposure to formaldehyde probably was extensive in our case patient, other factors may also be involved. Smoking should be considered a possible risk factor for maxillary sinus cancer. The patient had been a smoker of 10 sigarettes a day for 45 years. There is some evidence in support of an association between tobacco smoking and sinonasal cancer. However, in all the studies of concern, there was an increased risk for squamous-cell carcinomas, whereas the risk for adenocarcinomas and other types was generally not increased [17]. We therefore consider smoking a less likely etiological factor in our patient. The presence of primary Sjögren’s syndrome may also play a role. Elevated risk of malignancies has been associated with Sjögren’s syndrome. However, this has mainly been reported for lymfomas [18].

An association between formaldehyde exposure and work-related asthma among health care workers is well known [19]. Concerning cancer hazard, the levels and duration of exposure are assumed to be higher than what is necessary to induce asthma. Interestingly, the patient commented that her working conditions concerning formaldehyde exposure had improved markedly since she started working in the operating theatre in the 1960s. Still, we believe that there is reason for occupational physicians to remain alert to formaldehyde exposure as an occupational hazard among health care workers.

## Consent

Written informed consent was obtained from the patient for publication of this case report. A copy of the written consent is available by the Editor-in-Chief of this journal.
